# The first next-generation sequencing approach to the mitochondrial phylogeny of African monogenean parasites (Platyhelminthes: Gyrodactylidae and Dactylogyridae)

**DOI:** 10.1186/s12864-018-4893-5

**Published:** 2018-07-04

**Authors:** Maarten P. M. Vanhove, Andrew G. Briscoe, Michiel W. P. Jorissen, D. Tim J. Littlewood, Tine Huyse

**Affiliations:** 1 Department of Botany and Zoology, Faculty of Science, Masaryk University, Kotlářská 2, CZ-611 37 Brno, Czech Republic; 2 Zoology Unit, Finnish Museum of Natural History, University of Helsinki, P.O.Box 17, FI-00014 Helsinki, Finland; 3 Centre for Environmental Sciences, Research Group Zoology: Biodiversity & Toxicology, Hasselt University, Agoralaan Gebouw D, B-3590 Diepenbeek, Belgium; 4 Laboratory of Biodiversity and Evolutionary Genomics, Department of Biology, University of Leuven, Ch. Deberiotstraat 32, B-3000 Leuven, Belgium; 5 Biology Department, Royal Museum for Central Africa, Leuvensesteenweg 13, B-3080 Tervuren, Belgium; 6 Department of Life Sciences, Natural History Museum, Cromwell Road, London, SW7 5BD UK

**Keywords:** Cichlidae, Clariidae, *Cichlidogyrus*, Gene order, *Gyrodactylus*, *Macrogyrodactylus*, Mitogenome, Monogenea, Monopisthocotylea, Phylogenomics

## Abstract

**Background:**

Monogenean flatworms are the main ectoparasites of fishes. Representatives of the species-rich families Gyrodactylidae and Dactylogyridae, especially those infecting cichlid fishes and clariid catfishes, are important parasites in African aquaculture, even more so due to the massive anthropogenic translocation of their hosts worldwide. Several questions on their evolution, such as the phylogenetic position of *Macrogyrodactylus* and the highly speciose *Gyrodactylus*, remain unresolved with available molecular markers. Also, diagnostics and population-level research would benefit from the development of higher-resolution genetic markers. We aim to offer genetic resources for work on African monogeneans by providing mitogenomic data of four species (two belonging to Gyrodactylidae, two to Dactylogyridae), and analysing their gene sequences and gene order from a phylogenetic perspective.

**Results:**

Using Illumina technology, the first four mitochondrial genomes of African monogeneans were assembled and annotated for the cichlid parasites *Gyrodactylus nyanzae*, *Cichlidogyrus halli*, *Cichlidogyrus mbirizei* (near-complete mitogenome) and the catfish parasite *Macrogyrodactylus karibae* (near-complete mitogenome). Complete nuclear ribosomal operons were also retrieved, as molecular vouchers. The start codon TTG is new for *Gyrodactylus* and for Dactylogyridae, as is the incomplete stop codon TA for Dactylogyridae. Especially the *nad*2 gene is promising for primer development. Gene order was identical for protein-coding genes and differed between the African representatives of these families only in a tRNA gene transposition. A mitochondrial phylogeny based on an alignment of nearly 12,500 bp including 12 protein-coding and two ribosomal RNA genes confirms that the Neotropical oviparous *Aglaiogyrodactylus forficulatus* takes a sister group position with respect to the other gyrodactylids, instead of the supposedly ‘primitive’ African *Macrogyrodactylus*. Inclusion of the African *Gyrodactylus nyanzae* confirms the paraphyly of *Gyrodactylus*. The position of the African dactylogyrid *Cichlidogyrus* is unresolved, although gene order suggests it is closely related to marine ancyrocephalines.

**Conclusions:**

The amount of mitogenomic data available for gyrodactylids and dactylogyrids is increased by roughly one-third. Our study underscores the potential of mitochondrial genes and gene order in flatworm phylogenetics, and of next-generation sequencing for marker development for these non-model helminths for which few primers are available.

**Electronic supplementary material:**

The online version of this article (10.1186/s12864-018-4893-5) contains supplementary material, which is available to authorized users.

## Background

Ectoparasitic infections in bony fishes are dominated by monogeneans [1]. Among their most species-rich taxa are Gyrodactylidae and Dactylogyridae [2]. These include, respectively, the supergenera *Gyrodactylus* and *Dactylogyrus*, some of the most significant radiations of flatworm fish parasites [1]. Around 500 species of *Gyrodactylus* have been described at present ([3] and references therein), but the estimated species number is much higher [4]. These minute flatworms attach to their host by means of an opisthaptor, often used in monogenean taxonomy [5]. The resulting disruption of the epidermis may facilitate secondary infections by e.g. fungi or bacteria [6]. Some genera within these families, such as *Gyrodactylus*, *Macrogyrodactylus*, *Dactylogyrus* and *Cichlidogyrus* include fish pathogens, especially in captive-reared stocks and after anthropogenic co-introduction outside of their native range [2, 5, 7, 8]. In Africa, the most important aquaculture fishes are species of Cichlidae and Clariidae, including the Nile tilapia and the North African catfish, which have been introduced worldwide [9, 10]. These fish families are also relatively well-studied for monogenean parasites (e.g. [3, 11]). They harbour several originally African monogeneans that are widely distributed within and outside Africa, and that are important in the study of parasite ecology, evolution and invasion biology because of the economic and scientific importance of their hosts [12].

In view of the important threats that disease poses to the sustainable development of aquaculture in developing countries, a better monitoring and identification of aquatic pathogens is vital [13]. In Africa, better understanding of the diversity and ecology of fish parasites is needed to implement government policies on aquatic health management [14]. There is however a lack of monitoring, despite massive anthropogenic translocation of fishes that may lead to parasite co-introductions (e.g. [15]). Monogeneans, in particular, have been assessed as high-risk parasites in African aquaculture [16]. Since common procedures for the identification of these monogeneans are lethal to the host and require a high level of technical expertise, non-intrusive molecular diagnostics are called for (e.g. [17] for *Cichlidogyrus*). However, there is a lack of highly variable molecular markers for these animals [12].

In addition, the phylogenetic position of African monogenean lineages, including several endemic or recently discovered genera, is often poorly understood, also largely due to low phylogenetic coverage. For example, the currently most frequently used markers, situated in the nuclear ribosomal DNA region, have not fully resolved the position of the typically African *Macrogyrodactylus*. The representatives of this genus infect clariid catfishes, among other hosts [18, 19]. Malmberg [20] suggested, based on morphological data, that the genus comprises the earliest diverging lineage of gyrodactylids. This is a family of mainly viviparous monogeneans, although with some oviparous representatives [6]. However, mitogenomic phylogenetics recently suggested the Neotropical oviparous gyrodactylid *Aglaiogyrodactylus forficulatus* as sister to all other, viviparous, family members [21]. Also, Malmberg’s hypothesis was contradicted by nuclear phylogenetic data placing *Macrogyrodactylus* with other viviparous lineages [19]. Another long-standing issue in the phylogeny of this monogenean family, is the status of its most species-rich and well-studied genus, *Gyrodactylus* ([22] and references therein), first suggested to be paraphyletic by Kritsky & Boeger [23].

Recently, next-generation sequencing (NGS) approaches have facilitated marker development for non-model helminths [24]; this includes the assembly of mitogenomes for fish helminths [25, 26]. Here we want to apply this approach to the understudied, but highly diverse, African monogenean fauna. We targeted two common tilapia-infecting species of *Cichlidogyrus* (Dactylogyridae), the most speciose monogenean genus infecting African cichlid fishes [27]; one gyrodactylid parasite of cichlids; and a representative of *Macrogyrodactylus*. Through phylogenomic and gene order analysis, we address the following questions:

1. Are the Neotropical oviparous gyrodactylids still basal in a mitochondrial phylogeny when including the viviparous *Macrogyrodactylus*, which is supposedly the earliest divergent gyrodactylid lineage according to Malmberg [20]?
2. Does the phylogeny based on mitogenomic data confirm the paraphyly of *Gyrodactylus*?
3. Do the African representatives of Gyrodactylidae have the same gene order in their mitochondrial genome as the known Palearctic ones?
4. Do the African freshwater representatives of Dactylogyridae have the same gene order as seen in the only known dactylogyrid mitogenomes, from a Palearctic freshwater and an Indo-Pacific marine species?

## Results

**Fig. 1:**
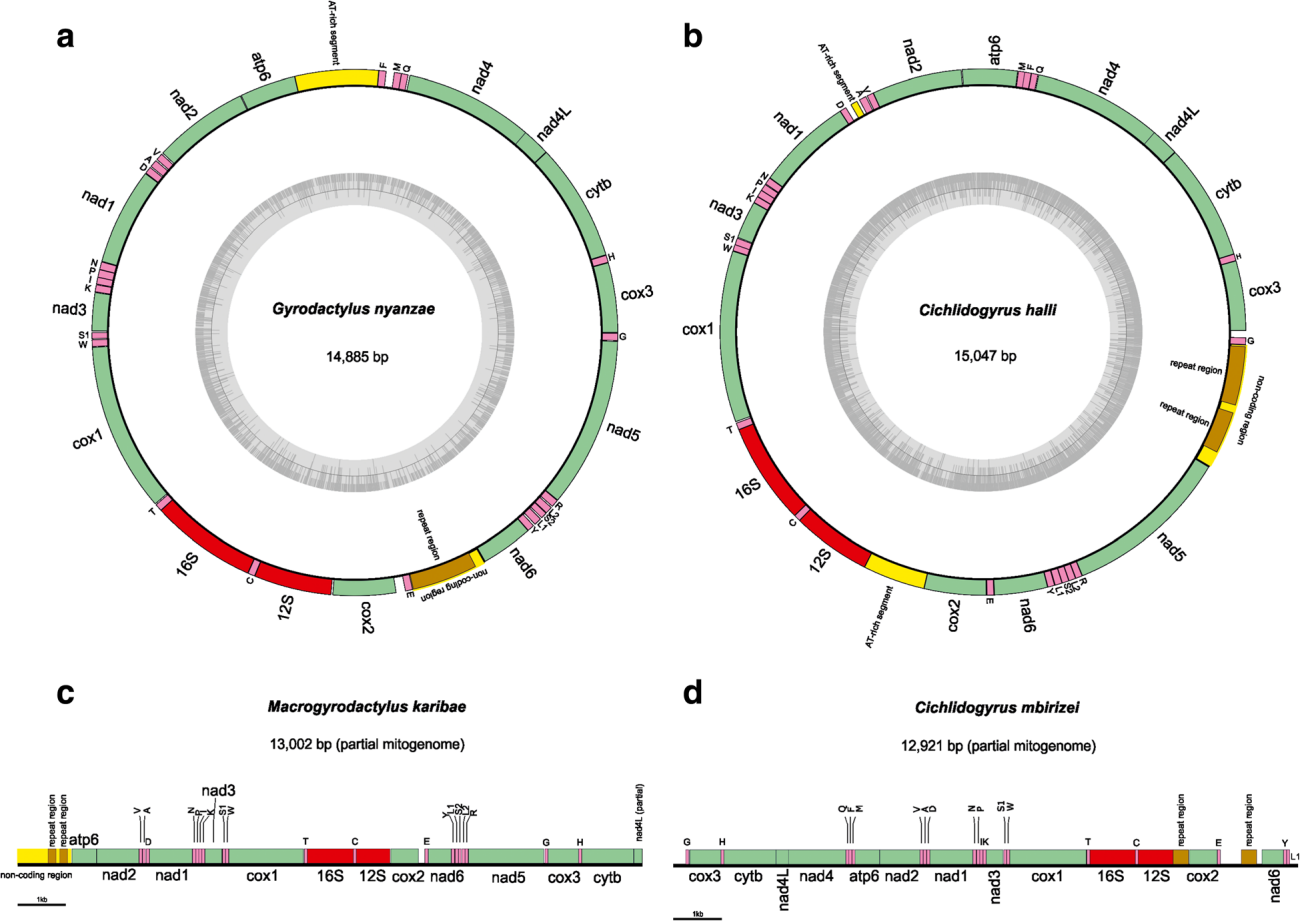
Mitochondrial genomes of four African monogeneans, including two members of the Gyrodactylidae: (**a**) *Gyrodactylus nyanzae*, (**c**) *Macrogyrodactylus karibae* (partial genome) and two representatives of the Dactylogyridae: (**b**) *Cichlidogyrus halli* and (**d**) *Cichlidogyrus mbirizei* (partial genome). The GC content is displayed for complete mitogenomes

Genomic DNA sequencing on three quarters of a MiSeq v. 3 flowcell yielded 15,980,972 indexed paired-end 300 bp reads. Complete mitochondrial genomes were assembled for *G. nyanzae* (with a length of 14,885 base pairs (bp)) and *C. halli* (15,047 bp). A circular genome could not be assembled for *C. mbirizei* (12,921 bp) and *M. karibae* (13,002 bp) (Fig. 1). The annotated sequences were deposited in NCBI GenBank under accession numbers MG970255-8. The total number of reads mapped across all of the assembled mitochondrial genomes was 12,776, accounting for 0.8 % of the genomic readpool obtained, with an average coverage of 160, 31, 76 and 42 reads for *G. nyanzae*, *C. halli*, *C. mbirizei* and *M. karibae*, respectively. The coverage along the various protein-coding and ribosomal RNA (rRNA) genes is detailed in Table 1. All complete protein-coding genes (PCGs) were represented by a minimum of 15× coverage, with a minimum average coverage of 29× (Table 1). The ribosomal operons of *G. nyanzae* (6799 bp), *M. karibae* (6675 bp), *C. halli* (7496 bp) and *C. mbirizei* (7005 bp) were deposited as additional molecular vouchers for these species, under NCBI GenBank accession numbers MG973075-8; their annotation is provided in Additional file 1. We did not include these sequences in our phylogenetic analyses because of the lack of published complete ribosomal operons for other species represented.

**Table 1:** Minimum–maximum and average coverage (in number of reads) of the protein-coding and rRNA genes for the four assembled mitochondrial genomes

	*Gyrodactylus nyanzae*		*Macrogyrodactylus karibae*		*Cichlidogyrus halli*		*Cichlidogyrus mbirizei*	
Gene	Range	Average	Range	Average	Range	Average	Range	Average
*cox*3	151–184	165	21–48	39	41–59	51	101–131	118
*cyt*b	136–174	155	16–44	29	51–74	61	115–165	139
*nad*4L	159–198	179	–	–	66–79	72	155–199	179
*nad*4	125–217	177	–	–	42–75	56	112–187	140
*atp*6	119–194	166	34–43	39	54–72	63	98–140	119
*nad*2	130–195	167	36–56	47	49–68	56	121–177	149
*nad*1	148–205	183	15–61	30	49–74	61	134–170	147
*nad*3	151–175	163	27–44	35	48–72	59	152–180	166
*cox*1	125–182	149	29–56	42	33–70	51	112–177	148
16S rRNA	102–140	122	22–44	34	39–87	61	157–315	263
12S rRNA	103–134	115	28–40	33	31–56	43	225–288	253
*cox*2	129–151	139	37–50	44	48–69	57	199–349	252
*nad*6	107–162	141	27–43	35	38–57	49	76–130	112
*nad*5	127–183	160	24–49	38	27–70	49	–	–

Averages are rounded to the nearest integer. “-”indicates a partially characterized or missing gene

### Mitogenome characterisation

The protein-coding, ribosomal RNA and tRNA genes are characterised in Table 2. The two complete mitogenomes were each comprised of 22 tRNA genes (including two for the amino acids serine and leucine each) and 12 intron-free PCGs and lack the *atp*8 gene. The genes coding for the large and small subunit of the mitochondrial rRNA were identified for all four species, as were most PCGs (Fig. 1). Only the *nad*5 gene of *C. mbirizei* and the *nad*4 gene and part of the *nad*4L gene of *M. karibae* were missing. Within the respective monogenean families, start and stop codons of most genes are conserved in these African species (Table 2). Within the two gyrodactylids, only the stop codons of the *cyt*b, *atp*6, *cox*1 and *nad*6 genes differ; within dactylogyrids, this is only the case for the genes coding for *cyt*b, *nad*3 and *cox*1. The only difference in start codon usage was found in the *nad*2 and *nad*6 gene in *Cichlidogyrus*. Abbreviated stop codons occur in the *cox*3 and *nad*2 genes of the two species of *Cichlidogyrus*.

**Table 2:** Overview of the length of markers, the start and stop codons (protein-coding genes) and anticodons (tRNA genes) for the four assembled mitochondrial genomes

	*Gyrodactylus nyanzae*				*Macrogyrodactylus karibae*			*Cichlidogyrus halli*				*Cichlidogyrus mbirizei*		
	Position	Length	Start/stop codon	Anticodon	Length	Start/stop codon	Anticodon	Position	Length	Start/stop codon	Anticodon	Length	Start/stop codon	Anticodon
*cox*3	1-639	639	ATG/TAA		639	ATG/TAA		13-658	646	ATG/T		646	ATG/T	
*trn*H	642-709	68		GTG	63		GTG	659-722	64		GTG	63		GTG
*cyt*b	713-1789	1077	ATG/TAA		1080	ATG/TAG		723-1799	1077	ATG/TAA		1077	ATG/TAG	
*nad*4L	1792-2049	258	ATG/TAA		171 (partial)	ATG/-		1803-2063	261	GTG/TAG		261	GTG/TAG	
*nad*4	2022-3221	1200	ATG/TAA		–	–		2036-3250	1215	ATG/TAG		1212	ATG/TAG	
*trn*Q	3237-3299	63		TTG	–		–	3256-3317	62		TTG	62		TTG
*trn*M	3298-3364	67		CAT	–		–	3374-3438	65		CAT	63		CAT
*trn*F	3437-3504	68		GAA	–		–	3318-3381	64		GAA	64		GAA
AT-rich segment	3505-4268	764			–			–	–			–		
*atp*6	4269-4781	513	ATG/TAA		513	ATG/TAG		3443-3952	510	ATG/TAG		510	ATG/TAG	
*nad*2	4788-5663	876	ATG/TAA		876	ATG/TAA		3960-4792	833	ATG/TA		833	GTG/TA	
*trn*V	5676-5741	66		TAC	63		TAC	4793-4855	63		TAC	64		TAC
*trn*A	5754-5823	70		TGC	64		TGC	4866-4934	69		TGC	68		TGC
AT-rich region	–	–	–	–	–	–	–	4958-5022	65					
*trn*D	5834-5899	66		GTC	65		GTC	5075-5136	62		GTC	64		GTC
*nad*1	5904-6791	888	GTG/TAA		888	GTG/TAA		5137-6024	888	GTG/TAG		888	GTG/TAG	
*trn*N	6796-6867	72		GTT	68		GTT	6027-6092	66		GTT	68		GTT
*trn*P	6868-6939	72		TGG	64		TGG	6098-6162	65		TGG	64		TGG
*trn*I	6938-7002	65		GAT	64		GAT	6162-6228	67		GAT	67		GAT
*trn*K	7007-7073	67		CTT	62		CTT	6230-6292	63		CTT	64		CTT
*nad*3	7078-7428	351	ATG/TAA		351	ATG/TAA		6294-6641	348	GTG/TAA		348	GTG/TAG	
*trn*S1	7441-7498	58		GCT	59		GCT	6648-6712	65		GCT	58		GCT
*trn*W	7506-7570	65		TCA	65		TCA	6710-6769	60		TCA	63		TCA
*cox*1	7575-9122	1548	ATG/TAA		1548	ATG/TAG		6773-8356	1584	ATG/TAG		1581	ATG/TAA	
*trn*T	9132-9199	68		TGT	63		TGT	8369-8433	65		TGT	63		TGT
16S rRNA	9200-10,170	971			959			8434-9373	940			939		
*trn*C	10,172-10,237	66		GCA	60		GCA	9374-9436	63		GCA	61		GCA
12S rRNA	10,239-10,948	710			706			9437-10,166	730			722		
AT-rich segment/repeat region	–	–	–	–	–	–	–	10,167-10,743	577			320		
*cox*2	10,963-11,538	576	TTG/TAA		579	TTG/TAA		10,744-11,316	573	ATG/TAA		576	ATG/TAA	
*trn*E	11,622-11,689	68		TTC	68		TTC	11,321-11,386	66		TTC	61		TTC
repeat region	11,694-12,296	603			–	–	–	–	–			320		
*nad*6	12,391-12,867	477	ATG/TAA		477	ATG/TAG		11,390-11,881	492	TTG/TAA		447	ATG/TAA	
*trn*Y	12,869-12,934	66		GTA	64		GTA	11,883-11,947	65		GTA	63		GTA
*trn*L1	12,951-13,021	71		TAG	66		TAG	11,951-12,018	68		TAG	63		TAG
*trn*S2	13,035-13,093	59		TGA	58		TGA	12,019-12,083	65		TGA	–		–
*trn*L2	13,097-13,163	67		TAA	68		TAA	12,084-12,148	65		TAA	–		–
*trn*R	13,176-13,246	71		TCG	64		TCG	12,150-12,212	63		TCG	–		–
*nad*5	13,250-14,806	1557	ATG/TAG		1566	ATG/TAG		12,214-13,758	1545	ATG/TAG		–	–	
repeat region					174			13,911-14,302	392			–		
repeat region					167			14,372-14,915	544			–		
*trn*G	14,812-14,880	69		TCC	64		TCC	14,931-14,996	66		TCC	64		TCC

Positions on the genome are only indicated for complete mitogenomes

Mitogenome gene arrangement differed between the African representatives of the dactylogyrids and gyrodactylids only in a single tRNA gene transposition. Protein-coding genes appeared in identical order (see below for pairwise gene order comparisons in a phylogenetic context). Several non-coding regions (NCRs) were observed in all four mitogenomes (Fig. 1). In *G. nyanzae*, one of them, a 603 bp stretch between the genes for *nad*6 and *trn*E, nearly perfectly repeats (except for one substitution) a fragment of 282 bp 2.1 times. The second one, an AT-rich segment (ca. 17% GC content) of 764 bp between the *atp*6 and *trn*F genes, was not identified as a repeat region. In contrast to this, and to the single repeat region of *G. nyanzae*, two consecutive repeat regions were identified adjacent to the *atp*6 gene in the partial mitogenome of *M. karibae*, one 174 bp long with a period of 87 bp (two repeats, 95% match) and the other one 167 bp long with a period of 73 bp (2.3 repeats, 99% match). It has to be noted however, that the possibility of a second, potentially longer non-coding region cannot be excluded due to the double amount of reads in this non-coding region. However, the annotation is incomplete and the exact location can only be inferred using conventional Sanger sequencing. Also the mitogenome of *C. halli* has two repeat regions, between the *trn*G and *nad*5 genes: a 392 bp fragment with repeats of 86 bp (4.6 repeats, 99% match), and a 544 bp fragment with repeats of 167 bp (3.3 repeats, 98% match). In addition, there are AT-rich segments between the *cox*2 and 12S rRNA genes (577 bp with a GC content of ca. 20%) and between the *trn*D and *trn*A genes (65 bp with a GC content of ca. 33%, displaying 58% sequence similarity with a motif in the former AT-rich segment). In the mitogenome of its congener *C. mbirizei*, a 320 bp stretch is duplicated (97% match) between the genes coding for *cox*2 and 12S rRNA on the one hand, and *nad*6 and *trn*E on the other hand.

**Fig. 2:**
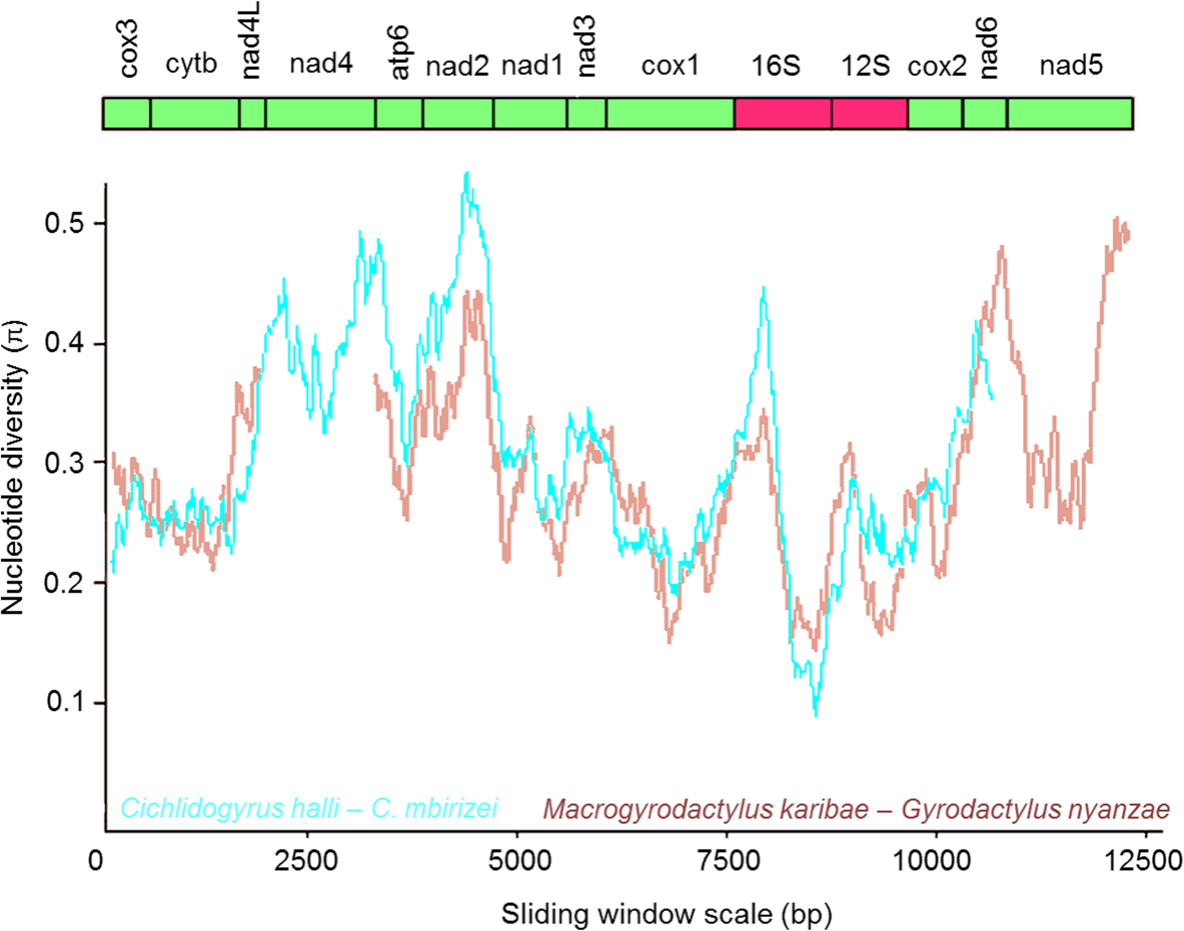
Sliding window analyses (window size 300 bp, step size 10 bp) of the alignment of mitochondrial protein-coding and ribosomal RNA genes used for the phylogenetic analyses of the four mitochondrial genomes of African monogeneans. The lines indicate the nucleotide diversity between two dactylogyrids (*Cichlidogyrus halli* and *C. mbirizei*, in blue) and two gyrodactylids (*Gyrodactylus nyanzae* and *Macrogyrodactylus karibae*, in red). Gene boundaries are indicated above the graph

**Fig. 3:**
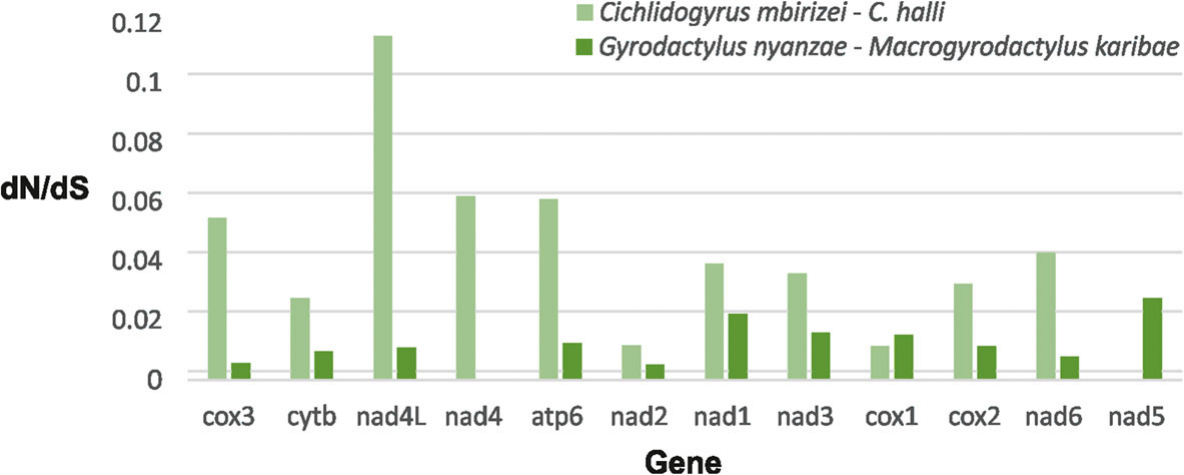
Ratio of non-synonymous to synonymous substitution rates for the protein-coding genes in two pairwise comparisons, between the mitogenomes of African dactylogyrid and gyrodactylid monogeneans, respectively. For *Macrogyrodactylus karibae*, no *nad*4 sequence was available, while the *nad*5 gene was lacking for *Cichlidogyrus mbirizei*

The sliding window analysis showed concurring patterns and similar values of nucleotide diversity across the mitochondrial genes for the gyrodactylid and dactylogyrid comparisons (Fig. 2). The highest values were found in the genes coding for subunits of NADH dehydrogenase. The dN/dS ratios in the two pairwise comparisons vary, with the highest values in genes coding for subunits of NADH dehydrogenase (Fig. 3). Values remain around or below 0.1 and are higher for the comparison between the two dactylogyrids than between the two gyrodactylids.

### Phylogenetic and gene order analyses

**Fig. 4:**
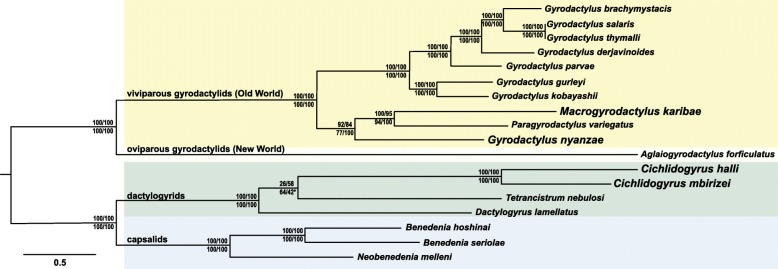
Midpoint-rooted maximum likelihood phylogram of monopisthocotylean monogeneans based on 12 protein-coding and two ribosomal RNA genes. Support values displayed from (above branch): Shimodaira-Hasegawa-like approximate likelihood ratio test/ultrafast bootstrap, both implemented in IQ-TREE, (below branch) bootstrap in RAxML/Bayesian inference (posterior probability) in MrBayes. An asterisk (*) indicates that this partition was not withheld in the Bayesian consensus tree; the clade grouping *Dactylogyrus lamellatus* and *Tetrancistrum nebulosi* as sister to a monophyletic *Cichlidogyrus* was supported by a posterior probability of 58%. Branch lengths indicate the expected number of substitutions per site

The concatenated alignment of 12 PCGs and two rRNA genes for 18 monogenean species contained 12,464 bp and 9184 variable sites, of which 8060 were parsimony-informative (although we do not analyse the data with parsimony). The topologies retrieved in ML and BI analyses were near-identical, except for the position of *Tetrancistrum nebulosi*; the resolution within Dactylogyridae is poor (Fig. 4). Capsalids and dactylogyrids firmly cluster together. *Macrogyrodactylus karibae* and *Paragyrodactylus variegatus* appear as sister taxa, albeit with long branches, presumably due to incomplete taxon coverage. *Gyrodactylus nyanzae* clusters with the clade of *Macrogyrodactylus* and *Paragyrodactylus*, rendering *Gyrodactylus* paraphyletic. *Aglaiogyrodactylus* is firmly positioned as basal to the other gyrodactylids.

**Fig. 5:**
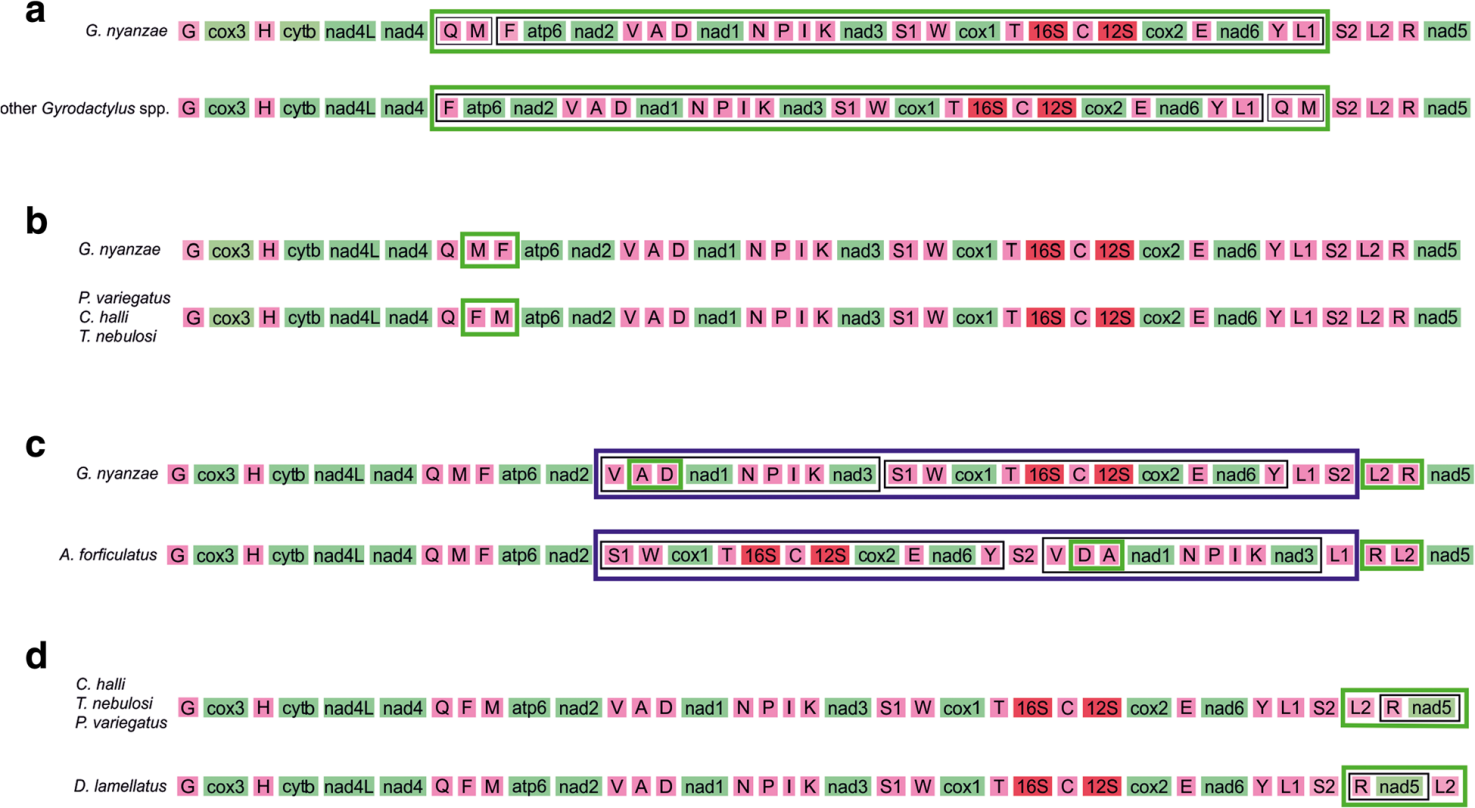
Family diagram explaining gene order changes between (**a**) African *Gyrodactylus nyanzae* and its Palearctic congeners (a single transposition), (**b**) *G. nyanzae* and *Paragyrodactylus variegatus* (a single transposition), (**c**) *G. nyanzae* and the Neotropical oviparous gyrodactylid *Aglaiogyrodactylus forficulatus* (two transpositions and a tandem duplication random loss event (TDRL)) and (**d**) *Dactylogyrus lamellatus* and the other dactylogyrids (a single transposition). Green boxes indicate transpositions, a dark blue box a TDRL. Only protein-coding genes, tRNA genes and rRNA genes of species for which a complete mitogenome was assembled, are shown

Within Gyrodactylidae, a transposition of two tRNA genes was the only difference in gene order between the African representatives and the Palearctic species of *Gyrodactylus* (Fig. 5a), while two adjacent tRNA genes were transposed between the African representatives and *P. variegatus* (Fig. 5b). The difference in mitochondrial gene order between the African gyrodactylids and the Neotropical *Aglaiogyrodacylus forficulatus* can be explained by a tandem duplication random loss (TDRL) event and two transpositions, or, alternatively, four transpositions (Fig. 5c). The gene order in the mitogenomes of both species of *Cichlidogyrus* was identical to that of their family member *T. nebulosi*, and the gyrodactylid *P. variegatus*. This gene order differed simply in one tRNA gene transposition from that of *Gyrodactylus nyanzae* (Fig. 5b) and from that of *Dactylogyrus lamellatus* (Fig. 5d).

## Discussion

As the low number of available genetic markers imposes limitations on research on non-model flatworms [28], improved and cost-efficient NGS offers ever-more opportunities for genomic work on helminths [29]. Using Illumina technology we assembled, for African gyrodactylid and dactylogyrid monogeneans, one complete and one partial mitogenome each (Fig. 1).

So far only nine gyrodactylid [21, 30–36] and two dactylogyrid [37, 38] monogenean mitogenomes have been published. Our study substantially increases the quantity of available mitogenomic data on these two most diverse monogenean families, by one-third, and offers the first mitogenomes from African representatives. The mitochondrial nucleotide diversity of monogeneans is aptly illustrated by the fact that universal barcoding primers for these species-rich helminths are unavailable [28]. Hence utilising NGS technologies is promising for monogeneans and for other non-model organisms for which typically few or no PCR primers are available. Newly obtained mitogenomes can provide a relatively large set of (coding) molecular markers for molecular evolutionary research. These can be used to develop taxon-specific mitochondrial primers for phylogeographic or population genetic analyses. Challenges however remain, such as the characterisation of AT-rich and repeat regions, in view of the read length of only 300 bp (see also [39]). Also, it is questionable to what extent this NGS approach is workable for rare or opportunistically collected monogenean species, as it has been applied mostly on pools of a considerable number of individuals (this study) or on single larger worm specimens (e.g. [39]). Furthermore, in view of frequent mixed infections, ideally specimens are morphologically identified prior to DNA extraction. This renders the pooling of specimens labour-intensive and sensitive to contamination. Developing reliable NGS shotgun methodologies that can work with single monogeneans, often very small (< 500–1000 μm) in length, will be a worthwhile goal for future molecular ecological and evolutionary studies.

### Mitogenome characterisation and potential for marker development

Throughout the PCGs in the four African mitogenomes, the typical start codons are mostly used: commonly ATG in gyrodactylids, and a combination of ATG and GTG in dactylogyrids. The same goes for the stop codons, typically TAA or TAG. Noteworthy exceptions are the *cox*2 gene of *G. nyanzae* and *M. karibae* and the *nad*6 gene of *C. halli*, with TTG as start codon. This has been reported in monogeneans before, e.g. in the *cox*2 gene of *Paragyrodactylus variegatus* [33]. However, it is reported for the first time here from a dactylogyrid monogenean [37, 38]; also, it is hitherto unique for a member of *Gyrodactylus*. It is somewhat unsurprising that the full breadth of codon usage diversity in this genus had not yet been captured, since existing mitogenomic data were limited to Palearctic species, all belonging to the subgenus *Limnonephrotus*, defined by Malmberg [40] on the basis of the excretory system. As regards abbreviated stop codons, the use of T had already been observed in a dactylogyrid monogenean, namely *Dactylogyrus lamellatus* [38]. The occurrence of TA as an incomplete stop codon, such as in the *nad*2 gene of both species of *Cichlidogyrus*, is newly reported for dactylogyrids. It has previously been reported in the same gene for *Gyrodactylus brachymystacis* [34].

Mitochondrial markers have a wide range of applications in micro-evolutionary and macro-evolutionary research on helminths. For most gyrodactylid and dactylogyrid monogeneans, a small set of established mitochondrial gene fragments (coding for *cox*1, *cox*2, *nad*2 and 16S rRNA) are the most variable markers available. These were applied in population genetics and demography [41, 42], in barcoding [43, 44], in phylogeography [45–48], to detect hybridisation [18] and to elucidate the phylogeny of closely related species [49] or genera [21, 50] or of higher-order taxa in monogeneans [51] and other flatworms (e.g. tapeworms [52]).

Within Palearctic gyrodactylids, *nad*2, *nad*4 and *nad*5 are the most variable genes in the mitochondrial genome and were therefore suggested as markers to study population-level processes [31, 34]. For African gyrodactylids and dactylogyrids, especially the *nad*2 gene seems promising for marker development as it is flanked by rather conservative stretches (Fig. 2). The dN/dS values for all mitochondrial PCGs fall well below 1 (Fig. 3), indicating purifying selection and confirming earlier mitogenomic work on monogeneans (e.g. [31, 38]). Overall purifying selection acting on mitochondrial genes has also been observed in a range of vertebrates [53, 54].

All hitherto known mitogenomes of species of *Gyrodactylus*, all representing the subgenus *Limnonephrotus*, contain two near-identical NCRs [34]. Conversely, such duplicated NCRs are absent in their congener *G. nyanzae* and, in our dataset, only found in *C. mbirizei*. Indeed, our results suggest substantial differences in the length, number and position of NCRs between African monogeneans even among gyrodactylids and within *Cichlidogyrus* (Fig. 1). There is no clear phylogenetic pattern, but a comparison with mitochondrial genomes of other gyrodactylid and dactylogyrid monogeneans indicates that non-coding (repeat) regions are commonly positioned in between certain pairs of genes: e.g. *trn*D and *trn*A in *C. halli* and *Aglaiogyrodactylus forficulatus* [21]; *trn*E and *nad*6 in *G. nyanzae* and *C. mbirizei*; *nad*5 and *trn*G in *C. halli* and *Tetrancistrum nebulosi* [37]; *trn*F and *atp*6 in *G. nyanzae* and its Palearctic congeners (e.g. [31, 34]); and 12S rRNA and *cox*2 in *C. halli* and *C. mbirizei*. Also a NCR containing two repeat regions in the vicinity of the *nad*5 gene, such as reported here for *C. halli*, has been reported before in dactylogyrids, namely by Zhang et al. [38] for *Dactylogyrus lamellatus*. Previous studies suggested the possibility of a functional role for certain NCRs [33] and the potential that NCRs offer for population-level research [38].

### Ribosomal operons and utility

Characterising full nuclear ribosomal operons provides a wealth of information for established and prospective molecular markers. Ribosomal DNA codes for all the nuclear ribosomal genes (18S, 5.8S and 28S rRNA) and also includes the external and internal transcribed spacer regions (ETS, ITS1, ITS2). As tandemly repeated units, ribosomal operons occur in high number, and the remarkable variation in rate of molecular evolution within and between nuclear rRNA gene regions has driven their popularity as a source for molecular markers in Metazoa [55] and within the parasitic flatworms [56]. Within flatworms ITS regions are popular for discriminating between closely related species [57], and complete 18S and partial (D1-D3) regions of 28S rDNA were used for phylogenetics of Monogenea (e.g. [58]). In combination with mitochondrial genes, nuclear ribosomal RNA genes are invaluable for discriminating hybrid species, especially important when revealing the identity of disease-causing parasites (e.g. [59]). Many nuclear rRNA gene regions have been used to discriminate species, to resolve phylogenetic relations and as molecular ecological markers amongst monogeneans [49, 60]. Within the newly characterised mitogenomes of African monogeneans in this study, the full operon ranged in size between 6675 and 7496 bp largely reflecting differences in length of spacer regions. We consider this to be a rich resource for a diversity of future studies, especially in the emerging field of environmental (eDNA) metabarcoding and metagenomics where access to highly conserved, and high copy number markers will greatly benefit accurate species identification [61]. In addition, a pairwise or multiple alignment of full ribosomal operons will readily highlight regions of sequence variability and conservation suggesting potential marker regions and regions for PCR primer design. Future studies aimed at population genetics, hybridisation, biogeography, cryptic species recognition, and host-parasite interactions will benefit from access to the full rRNA operon and the full mitogenomes of these, and additional taxa. Certainly, characterisation of full ribosomal operons by means of NGS genome skimming is considerably easier, and cheaper than by long PCR and primer walking using Sanger technology.

### Mitochondrial phylogeny, gene order and implications for the position of African gyrodactylid and dactylogyrid monogeneans

Our phylogenetic reconstruction based on 12 mitochondrial PCGs and 2 rRNA genes aimed to elucidate the position of African *Macrogyrodactylus*, *Gyrodactylus* and *Cichlidogyrus* (Fig. 4). All tree topologies firmly place the Neotropical oviparous *Aglaiaogyrodactylus forficulatus* as a sister lineage to all other representatives of Gyrodactylidae. This refutes Malmberg’s [20] hypothesis of *Macrogyrodactylus* being the most early divergent gyrodactylid. In addition, the inclusion of an African representative, *G. nyanzae*, renders *Gyrodactylus* paraphyletic. Hence, we provide the first mitochondrial data supporting the paraphyly of the genus, corroborating earlier phylogenetic hypotheses based on morphology [23] or nuclear rRNA genes [19, 22, 62].

The evolutionary distances and nucleotide diversity between the two representatives of *Cichlidogyrus* appear similar to, or even higher than, those between the two African gyrodactylids that are assigned to different genera, *Macrogyrodactylus* and *Gyrodactylus* (Figs. 2, 4). This corresponds to earlier work on these monogeneans that indicated the need for revision of *Cichlidogyrus* and *Gyrodactylus*. Vanhove et al. [62] and Přikrylová et al. [19] reported that genetic distances between gyrodactylid genera can reach the same order of magnitude as within the nominal genus *Gyrodactylus*, suggesting that a revision is necessary for several viviparous gyrodactylid genera including *Gyrodactylus*, although a monophyletic *Macrogyrodactylus* is strongly supported. Likewise, Pouyaud et al. [63] suggested that lineages within *Cichlidogyrus* sufficiently differ to be raised to generic status. In their analyses, the inclusion of *Scutogyrus* indeed rendered *Cichlidogyrus* paraphyletic, a finding confirmed in later analyses (e.g. [60]). The relationships between the only three dactylogyrid genera in the mitogenomic tree, all of them from the ‘Old World’, are not well resolved. Both *Cichlidogyrus* and *Tetrancistrum* have previously been mentioned as members of the Ancyrocephalinae (or Ancyrocephalidae). The monophyly of this (sub)family has often been challenged in earlier work (e.g. [50, 64, 65]). Two topologies (*Tetrancistrum* as a sister to *Cichlidogyrus* or, alternatively, to *Dactylogyrus*) have an equally low posterior probability under BI. Hence, our tree is not informative on the status of the Ancyrocephalinae versus the Dactylogyrinae, to which *Dactylogyrus* belongs. Although the polytomy makes it hard to favour either of the two alternative positions of *Tetrancistrum*, the gene order is identical between the representatives of *Tetrancistrum* and *Cichlidogyrus* in contrast to the representative of *Dactylogyrus*. We therefore consider the sister-group relation between the former two genera the biologically most likely hypothesis. This also corresponds to the nuclear rDNA-based results of Blasco-Costa et al. [66] suggesting that *Tetrancistrum* and *Cichlidogyrus* belong to the same clade of mostly marine ancyrocephalines. The affinity between *Cichlidogyrus* and marine genera, despite the likely sampling bias as many dactylogyrid genera have not yet been sequenced, is worth looking into because of the potential of cichlid parasites in elucidating the alleged role of marine dispersal in cichlid biogeography [67]. It would be worthwhile to consider mitochondrial gene order as a phylogenetic marker for further disentangling the relationships between purported dactylogyridean (sub)families.

While it is well-established that gene order is phylogenetically informative, it mainly seems to differ, certainly for PCGs, at the level of major flatworm lineages, such as between catenulids, triclads, polyclads and neodermatans [68]. Within the major flatworm clades, e.g. at order or family level, differences in mitogenome architecture mainly concern tRNA genes and NCRs (e.g. [69] for capsalids, [70] for triclads). This is confirmed in our results, where gene order differences within the dactylogyrids (Fig. 5d) and the viviparous gyrodactylids only concern tRNA genes (Fig. 5a, b). The transpositions seem to concur with evolutionary distance, e.g. simply two adjacent tRNA genes have swapped position within the *Macrogyrodactylus-Paragyrodactylus-Gyrodactylus nyanzae* clade (Fig. 5b). All viviparous gyrodactylids including *Macrogyrodactylus* have identical PCG orders. However, *Aglaiogyrodactylus forficulatus* displays a different PCG arrangement (Fig. 5c), which underscores its particular position within Gyrodactylidae, apart from the viviparous members of this family.

## Conclusions

The first mitogenomic data for African monogeneans are provided, characterising two partial and two complete mitochondrial genomes. These confirm earlier results on the variability of and purifying selection on mitochondrial genes in monogeneans, and highlight some patterns in the location of NCRs. These mitogenomes increased the known diversity of start and stop codon usage in dactylogyrids and in species of *Gyrodactylus*. A phylogeny based on 14 mitochondrial markers firmly confirmed the Neotropical oviparous *Aglaiogyrodactylus* as ‘basal’ to the other gyrodactylids, rather than the allegedly ‘primitive’ *Macrogyrodactylus*. Furthermore, it provided additional evidence for the paraphyly of *Gyrodactylus*. While the gene order for PCGs remained constant throughout the species considered, the study suggested tRNA transpositions to be phylogenetically informative for relationships within the family level.

As highlighted above, (mitochondrial) gene sequences are established tools in the identification of monogeneans, including potentially pathogenic and invasive strains of fish parasites, but their availability for African species remains limited. We hope that this study will contribute to marker development and diagnostics, and hence to ecological and evolutionary studies of African monogeneans.

## Methods

### Sampling

Fish hosts were collected in the Haut-Katanga province of the D.R. Congo in 2014. Sampling was carried out under research permit no. 863/2014 from the Faculté des Sciences Agronomiques of the Université de Lubumbashi, D.R. Congo. Two individuals of North African catfish *Clarias gariepinus* (vouchers URA 2014-P-1-004 at the Université de Lubumbashi and MRAC 2015–06-P tag AB49120835 at the Royal Museum for Central Africa (RMCA), Belgium) were caught in the Kiswishi River at Futuka Farm (11°29’S 27°39’E) on August 30th-31st and a hybrid between Nile tilapia *Oreochromis niloticus* and Mweru tilapia *Oreochromis mweruensis* (voucher MRAC 2015–06-P tag 2655) at the Kipopo station of the Institut National pour l’Etude et la Recherche Agronomiques (11°34’S 27°21’E) on August 27th. Hosts were sacrificed using an overdose of tricaine methanesulfonate (MS222). Parasites isolated either in situ or later from preserved fish gills were fixed and preserved in analytical-grade ethanol. Individual monogenean specimens were temporarily water-mounted between slide and coverslip, and identified on the basis of their morphology using keys and features described in [3, 18, 27]. Identified specimens were pooled per species in absolute ethanol: four specimens of *Macrogyrodactylus karibae* (supplemented with two extracts from [18]), 43 of *Cichlidogyrus mbirizei*, 18 of *Cichlidogyrus halli* and 44 of *Gyrodactylus nyanzae*. While *M. karibae* is a typical gill parasite of *Clarias gariepinus* known from southern Africa ([18] and references therein), *G. nyanzae* and especially *C. halli* are known from a wide range of cichlids throughout Africa [3, 27]. The two latter species have previously been reported from tilapias in the Haut-Katanga province [71]. *Cichlidogyrus mbirizei* was only recently described from the Lake Tanganyika endemic *Oreochromis tanganicae* [72]. It was afterwards also found on Nile tilapia and its hybrid *O. niloticus* x *mossambicus* [73, 74] and is here for the first time reported from *O. niloticus* x *mweruensis*. Both species of *Cichlidogyrus* have been co-introduced outside Africa, in nature and in aquaculture settings (e.g. [73–75]).

### DNA extraction and sequence assembly

Total genomic DNA was extracted using the DNeasy Blood and Tissue Kit (Qiagen) following the manufacturer’s instructions. The amount of double-stranded DNA isolated was measured with Qubit® 2.0 Fluorometer (Life Technologies, Paisley, UK) yielding 0.9 (*M. karibae*), 3.3 (*C. halli*), 3.2 (*C. mbirizei*) and 1.8 (*G. nyanzae*) ng/μl total DNA.

Samples for NGS were prepared and run at the DNA Sequencing Facility of the Natural History Museum, London, UK. Genomic DNA was indexed and libraries prepared using the TruSeq Nano DNA Sample Preparation Kit (Illumina, Inc., San Diego, USA), and run simultaneously on a MiSeq Illumina sequencer yielding 300 bp long paired-end reads. The new mitogenomes were directly assembled using Geneious v. 8.1.9 [76]. The sequences were first trimmed (error probability: 0.05, maximum ambiguity: 1) and then assembled. Partial *cox*1 sequences of *Gyrodactylus salaris* (NC008815 [30]) (for *G. nyanzae*), *Macrogyrodactylus clarii* (GU252718 [18]) (for *M. karibae*) and *Cichlidogyrus zambezensis* (KT037411 [49]) (for representatives of *Cichlidogyrus*) were used as reference sequence to extract *cox*1 reads from the Illumina genomic readpool to form the consensus sequence to subsequently map the reads on successive iterations. Trimmed reads were mapped back to the contigs in order to estimate the full mitochondrial genome coverage, trim the overlapping regions to create a circular molecule, and to inspect for potential mapping/assembly errors in problematic regions such as repetitive regions [77]. In instances where disagreements occurred between reads, the consensus sequence was generated by choosing the most frequently represented base.

Using nuclear ribosomal RNA gene sequences for *Cichlidogyrus halli* and *Macrogyrodactylus congolensis* from GenBank (accessions: HE792784 [60] and HF548680 [19] respectively), fragments of the ribosomal RNA operon were identified and assembled using the same iterative process as described for the mitochondrial genome. Exact coding positions of the 18S and 28S nuclear rDNAs, as well as the respective 5′ and 3′ boundaries of the external transcribed spacers, were determined using RNAmmer [78]. Subsequently the complete annotation was compared with the fully-annotated human rDNA repeating unit (GenBank accession: HSU13369).

### Mitogenome annotation

The identity and boundaries of individual PCGs and rRNA genes were determined using the MITOS web server [79] in combination with the visualisation of open reading frames in Geneious and a comparison with alignments of available mitogenomes of closely related monopisthocotylean monogeneans. In addition to MITOS, the ARWEN v. 1.2 [80] and tRNAscan-SE v. 2.0 [81] web servers were used to identify the tRNA-coding regions. When results between applications conflicted, the solution proposing a 7 bp acceptor stem was chosen. We checked for repeat regions with Tandem Repeats Finder [82] and YASS [83]. The resulting mitogenomes were visualised in OGDRAW v. 1.1 [84].

### Alignment, sequence analysis, phylogenetic reconstruction and gene order analysis

**Fig. 6:**
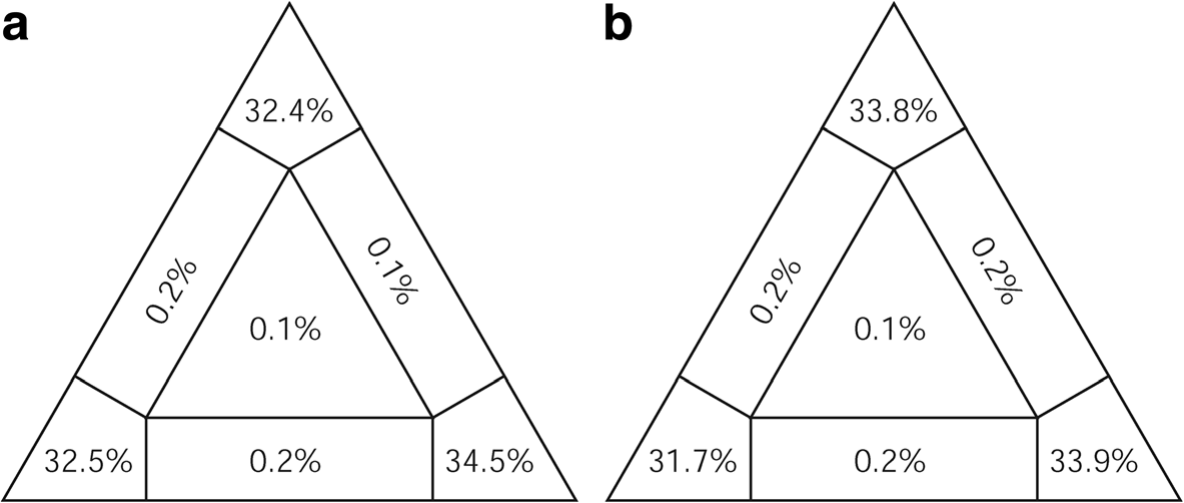
Likelihood mapping (**a**) before and (**b**) after Gblocks trimming, demonstrating the high phylogenetic content and suggesting there is no need for alignment cleaning in the case of this dataset

Ribosomal RNA genes were aligned by MAFFT v. 7 [85] using the Q-INS-i iterative refinement method, taking into account RNA secondary structure [86]. Codon-based alignment of all obtained PCGs was performed under the echinoderm and flatworm mitochondrial genetic code [87] using MUSCLE [88] implemented in SeaView v. 4.6.2 [89]. Since omitting unreliable portions of the alignment may increase resolution in phylogenomic reconstructions [90], an alternative alignment was obtained by trimming in Gblocks v. 0.91b [91], implemented for the PCGs in TranslatorX [92], carrying out codon-based MAFFT alignment followed by alignment cleaning in Gblocks. Options for a less stringent selection were selected, allowing smaller final blocks, gap positions within the final blocks, and less strict flanking positions. Especially for smaller datasets, trimming entails the risk of removing information contributing to phylogenetic signal [90]. Therefore, likelihood mapping [93] was performed in TREE-PUZZLE v. 5.3 [94] to compare the phylogenetic content of the complete and trimmed concatenated alignment. The percentage of fully, partially and unresolved quartets was 99.4, 0.5 and 0.1 in both cases, hence trimming did not increase phylogenetic content and the original alignment was preferred for downstream analyses (Fig. 6). Comparing, in DAMBE [95], the index of substitution saturation with its critical value at which sequences would start to fail to recover the true phylogeny, indicated little substitution saturation for this dataset [96].

Using the aligned sequences, two pairwise comparisons between members of the same monogenean family (*C. halli* versus *C. mbirizei*; *G. nyanzae* versus *M. karibae*) were made. Firstly, we visualised the nucleotide diversity by a sliding window analysis of nucleotide diversity (π) in DnaSP v. 5.10.01 [97], with a window size of 300 bp and a step size of 10 bp. To allow comparison between the dactylogyrid and gyrodactylid haplotypes, this approach was limited to the PCGs and rRNA genes. Secondly, for the PCGs of the same pairs of species, the proportion of non-synonymous versus synonymous substitutions (dN/dS ratio) was calculated in the codeml program of PAML [98] as implemented in PAL2NAL [99].

To situate the African monogeneans under study within their respective families, the PCGs and rRNA genes of all available dactylogyrid [37, 38] and gyrodactylid [21, 30–36] mitogenomes were included in phylogenetic analyses. The species of Capsalidae for which mitogenomes are available [69, 100, 101] were also included as they strongly cluster with the dactylogyrids [21, 38].

The best partition scheme and the optimal models of molecular evolution were determined based on the Bayesian Information Criterion using ModelFinder [102] with partition merging [103]. The selected partitions and models are shown in Table 3. These were used for Bayesian inference (BI) of phylogeny, whereby posterior probabilities were calculated in MrBayes v. 3.2 [104] over 10 million generations, sampling the Markov chain at a frequency of 100 generations. Chain stationarity was evidenced by a standard deviation of split frequencies of 8.10^–4^, absence of a trend in the probabilities plotted against the generations, and a potential scale reduction factor [105] converging towards 1. One-fourth of the samples were discarded as burn-in. The same partitions were used in a maximum likelihood (ML) search in IQ-TREE [106], using four gamma-rate categories and an edge-linked partition model. Nodal support was assessed through 10,000 ultrafast bootstrap [107] and 1000 Shimodaira-Hasegawa-like approximate likelihood ratio test [108] replicates. In addition, a ML tree was constructed in RAxML v. 8.1.21 [109] implemented in raxmlGUI v.1.3 [110], using codon-specific partitions under the GTR + Γ + I model with joint branch length optimization, and with 1000 bootstrap samples to calculate support values. ALTER [111] and GenBank 2 Sequin [112] were used for file conversion, and SequenceMatrix [113] to concatenate alignment files.

**Table 3:** Best partition scheme for the dataset of two ribosomal RNA genes and 12 protein-coding genes in the mitochondrial genomes of 14 monopisthocotylean monogenean flatworms

Partition	Model of molecular evolution
12S + 16S + first codon positions of *atp*6, *nad*2, *nad*3, *nad*4, *nad*4L, *nad*5, *nad*6	TIM2 + I + G
second codon positions of *atp*6, *nad*1, *cox*1, *cox*2, *cox*3, *cyt*b	TVM + I + G
third codon positions of *atp*6, *nad*1, *nad*2, *nad*3, *nad*4, *nad*4L, *nad*5, *nad*6, *cox*1, *cox*2, *cox*3, *cyt*b	TIM2 + I + G
first codon positions of *nad*1, *cox*1, *cox*2, *cox*3, *cyt*b	TIM2 + I + G
second codon positions of *nad*2, *nad*3, *nad*4, *nad*4L, *nad*5, *nad*6	TVM + I + G

The number of gamma rate categories was set to four

Gene orders were compared, and family diagrams of gene orders constructed, using CREx [114]. For those genes that were available from the partial mitogenomes, gene order was identical between *M. karibae* and *G. nyanzae*, and between *C. mbirizei* and *C. halli*, respectively. Therefore, only the complete mitogenomes could be included in gene order analyses. For the same reason of comparability, non-coding regions (NCRs) were omitted in gene order analysis.

## Additional file

Additional file 1: **Table S1.** Annotation of the ribosomal operons of the four African monogenean species. (TXT 1 kb)

## Abbreviations

BI: Bayesian inference
bp: Base pairs
MITObim: Mitochondrial baiting and iterative mapping
ML: Maximum likelihood
NCR: Non-coding region
NGS: Next-generation sequencing
PCG: Protein-coding gene
rDNA: Ribosomal DNA
rRNA: Ribosomal RNA
TDRL: Tandem duplication random loss event
